# Prognostic and chemotherapeutic response prediction by proliferation essential gene signature: Investigating POLE2 in bladder cancer progression and cisplatin resistance

**DOI:** 10.7150/jca.93023

**Published:** 2024-02-04

**Authors:** Liying Yu, Na Lin, Yan Ye, Shuang Zhou, Yanjuan Xu, Jiabi Chen, Wei Zhuang, Qingshui Wang

**Affiliations:** 1 Central Laboratory, the Second Affiliated Hospital of Fujian Medical University, Quanzhou 362000, China.; 2 Department of Pathology, the Second Affiliated Hospital of Fujian Medical University, Quanzhou 362000, China.; 3 Ganzhou Key Laboratory of Molecular Medicine, the Affiliated Ganzhou Hospital of Nanchang University, Ganzhou, Jiangxi, 341000, China.; 4 The Second Clinical Medical School of Fujian Medical University, Quanzhou, Fujian Province, 362000, China.; 5 Department of Urology, the Second Affiliated Hospital of Fujian Medical University, No. 34 Zhongshan North Road, Quanzhou 362000, Fujian.; 6 The Second Affiliated Hospital of Fujian University of Traditional Chinese Medicine, Fujian-Macao Science and Technology Cooperation Base of Traditional Chinese Medicine-Oriented Chronic Disease Prevention and Treatment, Innovation and Transformation Center, Fujian University of Traditional Chinese Medicine, Fuzhou, 361000, China.

**Keywords:** bladder cancer, POLE2, proliferation, tumor promoter, CRISPR-Cas9, bioinformatic analysis

## Abstract

**Background:** Bladder cancer (BLCA) is the most common genitourinary malignancy. Proliferation essential genes (PEGs) are crucial to the survival of cancer cells. This study aimed to build a PEG signature to predict BLCA prognosis and treatment efficacy.

**Methods:** BLCA PEGs and differentially expressed PEGs were identified using DepMap and TCGA-BLCA datasets, respectively. Based on the prognostic analysis of the differentially expressed PEGs, a PEG model was constructed. Subsequently, we analyzed the relationship between the PEG signature and prognosis of BLCA patients as well as their response to chemotherapy. Finally, we performed random forest analysis to target and functional experiments to validate the most significant PEG which is associated with BLCA progression. CCK-8, invasion, migration, and chemosensitivity assays were performed to assess effects of gene knockdown on BLCA cell proliferation, invasion and migration abilities, and cisplatin chemosensitivity.

**Results:** We screened 10 prognostic PEGs from 201 differentially expressed PEGs and used them to construct a PEG signature model. Patients with high PEG signature score (PEGs-high) exhibited worse OS and lower sensitivity to chemotherapy than those with PEGs-low. We also found significant correlations between the PEG score and previously defined BLCA molecular subtypes. This suggests that the PEG score may effectively predict the molecular subtypes which have distinct clinical outcomes. Random forest analysis revealed that POLE2 (DNA polymerase epsilon subunit 2) was the most significant PEG differentiating BLCA tissue and normal tissue. Bioinformatic analysis and an immunohistochemistry staining assay confirmed that POLE2 was significantly up-regulated in tumor tissues and was associated with poor survival in BLCA patients. Moreover, POLE2 knockdown inhibited the ability of cell clone formation, proliferation, invasion, immigration and IC50 of cisplatin.

**Conclusion:** The PEG signature acts as a potential predictor for prognosis and chemotherapy response in BLCA patients. POLE2 is a key PEG and plays a remarkable role in promoting the malignant progression and cisplatin resistance of BLCA.

## Introduction

BLCA is the most common genitourinary malignancy and one of the ten most common cancer types worldwide 1. It accounts for an estimated 570 000 new cases (3.0% of all cancer cases) and 210 000 deaths (2.1% of all cancer deaths) each year 1, 2, 3, 4. Approximately 70%-75% of BLCA are non-muscle invasive bladder cancer (NMIBC) which is easy to recur despite a high long-term survival rate after treatment. The remaining 25%-30% of BLCA cases belong to muscle-invasive bladder cancer (MIBC) which has a high degree of malignancy and a low five-year survival rate 5, 6, 7. Treatments for BLCA include surgery, intravesical infusion therapy, radiotherapy, targeted therapy, and immunotherapy. Although these treatments can control most types of BLCA cells, they are not ineffective for invasive or metastatic bladder tumors. These are partly due to an insufficient understanding of the biological mechanisms underlying BLCA recurrence and progression and lacking efficient biomarkers for the prediction of prognosis and treatment efficacy.

Molecular subtyping provides opportunities for precision medicine in BLCA 8. Numerous BLCA molecular subtype systems, such as the TCGA and Consensus systems, have been was established using RNA sequencing data 9, 10. For example, prognostic models based on DNA methylation-driven 11, epithelial-mesenchymal transition 12, and tumor microenvironment 13 related gene have been derived and shown to effectively predict overall survival and clinical outcomes of BLCA patients. There gene signatures provide novel insights into tumor progression and therapeutic strategies. Proliferation essential genes (PEGs) are critical for cancer cell growth. It has been shown that proliferation gene signatures can predict survival in some cancers, such as mantle cell lymphoma 14, prostate cancer 15. However, little is known about the clinical roles of PEGs specifically in BLCA. Given the heterogeneity of BLCA, further characterizing BLCA PEGs could advance molecular stratification and improve prognostication to aid treatment decision-making.

Genome-scale CRISPR-Cas9 16, 17 and loss-of-function RNA interference (RNAi) 18, 19 are powerful high-throughput methods to screen genes required for the survival and proliferation of cancer cells. The DepMap database integrates the CRISPR-Cas9 and RNAi-based knockout for genes across various cell lines 20. Simultaneously, a computation method, CERES, was developed to estimate gene-dependency levels from CRISPR-Cas9 essentiality screens 21. The CERES reflects gene importance for cell survival or proliferation which is affected by genotype, gene expression, and lineage of cell lines 22. The CERES scores of 0 indicate a median effect of nonessential genes, while -1 represents the median effect of common core essential genes 21. Identification of genes specifically essential in a few cell lines will be better suitable for drug targets as their lower probability to cause toxicity in noncancerous tissues under function-inhibiting conditions.

In this study, we carried out a systematic characterization of essential genes for the survival of BLCA cells based on CRISPR-datasets from Depmap, and gene expression datasets from the Tumor Cancer Genome Atlas (TCGA) and Gene Expression Omnibus (GEO). A PEG signature model was developed to quantify molecular subtypes in the BLCA. The PEG signature serves as an effective prognostic biomarker for accurately predicting the response of BLCA to chemotherapy treatment. Meanwhile, comprehensive bioinformatic analyses revealed that POLE2 was the most important PEG associated with BLCA progression and was up-regulated both in mRNA and protein levels in tumor tissues. We also assess the effects of POLE2 knockdown on cell proliferation, colony formation, invasion and migration ability, and chemosensitivity in BLCA cells. This study provides new insights into the development and prognosis prediction of BLCA.

## Materials and Methods

### Data source

The expression data of RNA-sequencing (RNA-seq) and the corresponding clinical information of BLCA patients (n=427) were obtained from the TCGA databases and were used to identify genes associated with survival. Three independent RNA-seq data of BLCA patients were downloaded from the GEO database and applied for validation, including GSE13507 (n =256), GSE32894 (n=308), and GSE31684 (n =93). Additionally, five GEO datasets of GSE13507 (n =233), GSE37851 (n =24), GSE40335 (n =24), GSE52519 (n =12), and GSE65635 (n =12) were used to profile POLE2 gene expression.

### Identification of essential genes for the development of BLCA

The DepMap portal (https://depmap.org/portal/) 23, 24, which is developed by the Broad Institute to facilitate the selection of cancer therapeutic targets, offers an assessment of gene dependency over 700 cancer cell lines, along with other information including gene profiling, gene copy number, and gene mutation status. Genes with amplified copy numbers can cause severe DNA damage during CRISPR-Cas9 cleavage and lead to cell growth arrest or apoptosis, which can lead to false positives. Therefore, accounting for gene copy number and single guide RNA (sgRNA) loss, a computational method decreasing the false-positive results, CERES, was developed to estimate gene-dependency levels from CRISPR-Cas9 essentiality screens 24. A negative CERES score indicates that knocking out the gene inhibits cell survival and proliferation. The more negative the CERES value, the greater the effect on survival and proliferation of the cell line after gene knockout. We identified BLCA-dependent genes based on the CERES score from the Depmap database. Essential genes were defined as genes with CERES scores <-1 in more than 75% of BLCA cell lines 25.

### PEG subtype profiling and comparison

Consensus clustering analysis was performed using the “ConsensusClusterPlus” package and classified BLCA patients into two distinct subtypes based on the expression levels of PEGs. Principal component analysis (PCA), gene set enrichment analysis (GSEA), and differences in prognosis of BLCA patients between the two subtypes were examined. The proportions of immune cell subsets were determined by the CIBERSORT algorithm and compared between the two subtypes.

### Construction of a prognostic PEG signature for BLCA

To identify BLCA PEGs with prognostic significance, a univariate Cox analysis was performed based on the TCGA dataset. Genes with a *p*-value < 0.05 were used for further screening of those with a prognostic signature using the Least Absolute Shrinkage and Selection Operator (LASSO) Cox regression analysis. LASSO utilizes the L1 regularization to make the coefficients of weak features turn to zero during fitting. Only features with non-zero coefficients were used in the model since we considered features with zero coefficients to be redundant. The genes with non-zero coefficients were defined as the final prognostic genes and used to construct a PEG risk score model: (coefficient of gene 1 × expression of gene 1) + (coefficient of gene 2 × expression of gene 2) + …. + (coefficient of gene n × expression of gene n).

### Validation of the prognostic model and genomic feature analysis

Based on the PEG risk score model, the risk score of each patient in the TCGA cohort was calculated. Patients were divided into the PEGs-high group and PEGs-low risk groups using the median risk score as a cutoff value. The performance of the signature was evaluated using Kaplan-Meier survival analysis. The same method was applied to other three independent validation datasets from GEO (GSE13507, GSE32894 and GSE31684) to validate the prognostic signature. Tumor mutational burden (TMB) was a novel therapeutic metric for determining immunotherapy sensitivity. The somatic mutation data of BLCA patients was also downloaded from the TCGA database. We calculated the tumor mutation burden, including somatic coding, base replacement, and insert-deletion mutations, based on the somatic mutation data using the “maftools” R package. The mutation type and frequency and TMB difference were compared between the PEGs-high and PEGs-low groups. PEG signature scores between different cancer grades or stages were also compared.

### Differential gene expression and functional enrichment analysis

Differential gene expression analysis was conducted using the “limma” R package. A volcano plot was used to display differentially expressed genes (DEGs). DEGs were defined as genes with *p*-value < 0.05 and |FoldChange| ≥ 1.5. The Kyoto Encyclopedia of Genes and Genomes (KEGG) functional enrichment of DEGs was performed by the “ClusterProfiler” R package. The KEGG pathways with *p*-value < 0.05 were considered significantly enriched.

### Drug sensitivity evaluation

To find potential molecular compounds for targeted therapy, we assessed the drug sensitivity of the PEGs. The Cancer Therapeutics Response Portal (CTRP) database (https://portals.broadinstitute.org/ctrp.v2.1/) was used for drug sensitivity analysis.

### Clinical cohort recruitment and sample collection

A cohort of 20 BLCA patients was recruited from the year of 2022 to 2023. The cancer tissue and paired paracancerous tissue were collected. The samples were provided by the Second Affiliated Hospital of Fujian Medical University (Quanzhou, Fujian, China) with the approval of the institutional research board and the donors' consent. Procedures followed in this study were under the ethical standards of concerned institutional policies (NO. 414/ Year 2022).

### Immunohistochemistry (IHC) staining analysis

We carried out an IHC staining assay to detect the protein expression level of POLE2 in BLCA tissues and normal tissues according to the standard immunoperoxidase staining procedure. Slides were incubated with anti-POLE2 antibodies (21146-1-AP, Proteintech, Wuhan, China, diluted 1:400) and then assessed by two pathologists. The percentage of positive tumor cells and staining intensity were scored by the two pathologists for each case, by which a multiplied result was obtained as the final IHC staining score. Four grades that ranged from 1 to 4 representing the percentage of stained-positive cells were evaluated: 1: 0-25%; 2: 26-50%; 3: 51-75%; and 4: 75-100%. The staining intensity was also defined as four grades: 0 for no staining, 1 for weak staining, 2 for moderate staining, and 3 for strong staining.

### Cells Culture

The BLCA cell line T24 was obtained from American Type Culture Collection (ATCC). T24 cells were cultured in DMEM medium containing 10% fetal bovine serum and 1% penicillin/streptomycin sulfate at 37°C and 5% CO_2_.

### CCK-8 Assay

T24 cells were plated in 96-well plates at a density of 2 × 10^4^ cells per well and cultivated for 24, 48, and 72 hours. A 10 μL CCK-8 solution was added four hours before absorbance measuring. After two hours of incubation at 37°C, the absorbance was measured with a microplate reader at 450 nm.

### Invasion assay

T24 cells were seeded in an invasion chamber with a serum-free medium. The underlying chamber was added with a complete medium and cultured for 48 h at 37°C, 5% CO_2_. The invaded cells passing through the membrane were fixed with methanol and stained with crystal violet for 10 min. Cells on the overhead chamber surface were wiped off with cotton swabs, and the number of invasive tumor cells was randomly photographed at six spots.

### Migration assay

T24 cells were cultured and aggregated to about 90%, and subsequently washed with PBS to remove the dislodged cellular debris. A 10 µL spear was used to make scratches. Cells were then cultured in serum-free medium for 48 h before being re-filmed under a microscope.

### Chemosensitivity assay

Cisplatin's chemosensitivity was assessed via the methyl thiazolyl tetrazolium method. Treatment on cells was conducted using varying concentrations of cisplatin (Sigma-Aldrich, St. Louis, Missouri, USA), ranging from 0 to 2.5 μg/ml for a continuous period of 48 hours. Subsequent calculations of the Inhibitory Concentration 50% (IC50) values were performed by utilizing GraphPad Prism 8 software. The data presented was drawn from three distinct experiments to support its reliability and consistency.

### RNA extraction and quantification

Total RNA from the T24 cells was extracted with TRIzol (Invitrogen, CA, USA), and was reverse transcript with an mRNA reverse transcription kit (Takara, Japan). Specific primers synthesized by Sangon Biotech (Shanghai, China) were used for RT-qPCR to detect the mRNA expression of POLE2. The following primer sequences were used: POLE2-forward primer 5'-TGAGAAGCAACCCTTGTCATC-3' and POLE2-reverse primer 5'-TCATCAACAGACTGACTGCATTC-3'. GAPDH primers; GAPDH-forward primer 5'-GCGGGGCTCTCCAGAACATCAT-3' and GAPDH-reverse primer 5'-CCAGCCCCAGCGTCAAAGGTG-3'. The relative expression of genes was quantified using the 2-^ΔΔCt^ method.

### Analysis of the correlation between POLE2 expression and stemness

Cancer stemness refers to the stem-cell-like phenotype of cancer cells which are known for their ability to self-differentiation, proliferation, and renewal 26. The stemness can be evaluated by RNA stemness score (RNAss) based on mRNA expression 27. To evaluate the POLE2 effect on stemness, the correlation between POLE2 expression and RNAss was examined using Spearman rank-based testing.

### Statistical Analysis

For pairwise comparisons, Wilcoxon signed rank-sum test was utilized. Two-way ANOVA was used to analyze the CCK8 assay. All *p* values < 0.05 were considered statistically significant.

## Results

### Identification of BLCA PEGs

**Figure 1:**
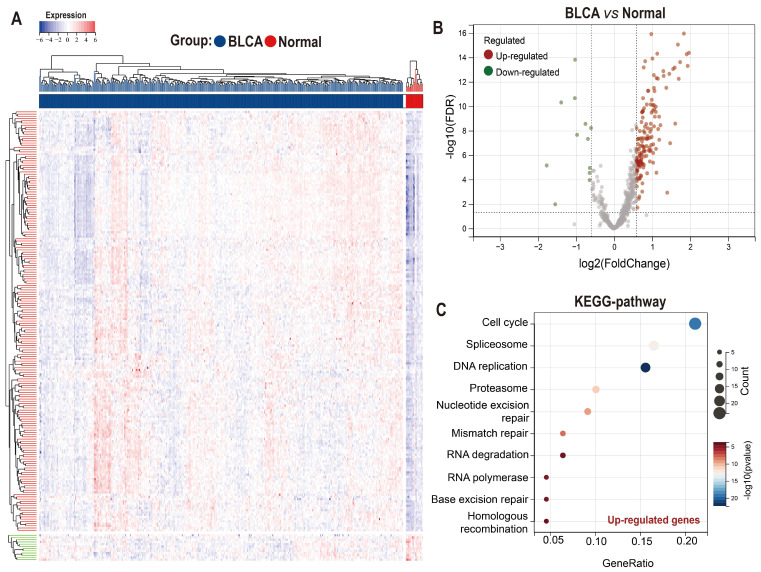
** Expression and functional enrichment of proliferation essential genes (PEGs) in the BLCA.** (A) Heat map and (B) volcano plot showing the differential expression of the 699 PEGs in BLCA patients from the TCGA-BLCA dataset. (C) KEGG enrichment of the 189 significantly up-regulated PEGs in the BLCA.

Taking advantage of the Achilles project of Depmap which aims to screen survival-related genes for various kinds of tumor cell lines using genome-scale CRISPR/Case 9, we identified 699 genes with CERES score < -1 in ≥ 75% BLCA cell lines (n=26) as potential PEGs for BLCA (Table S1). Among the 699 genes, 201 genes were differentially expressed between BLCA tissues and normal tissues in the TCGA-BLCA dataset (Figure 1A-B; Table S2). And 189 (94%) DEGs were significantly up-regulated while only 12 (6%) DEGs were down-regulated in BLCA tissues. KEGG functional enrichment showed that the enhanced DEGs involve in the cell cycle and genetic information processing including spliceosome, DNA replication, proteasome, nucleotide excision repair, mismatch repair, RNA degradation, RNA polymerase, Base excision repair, and homologous recombination (Figure 1C).

### Characterization of PEG subtypes in BLCA

**Figure 2:**
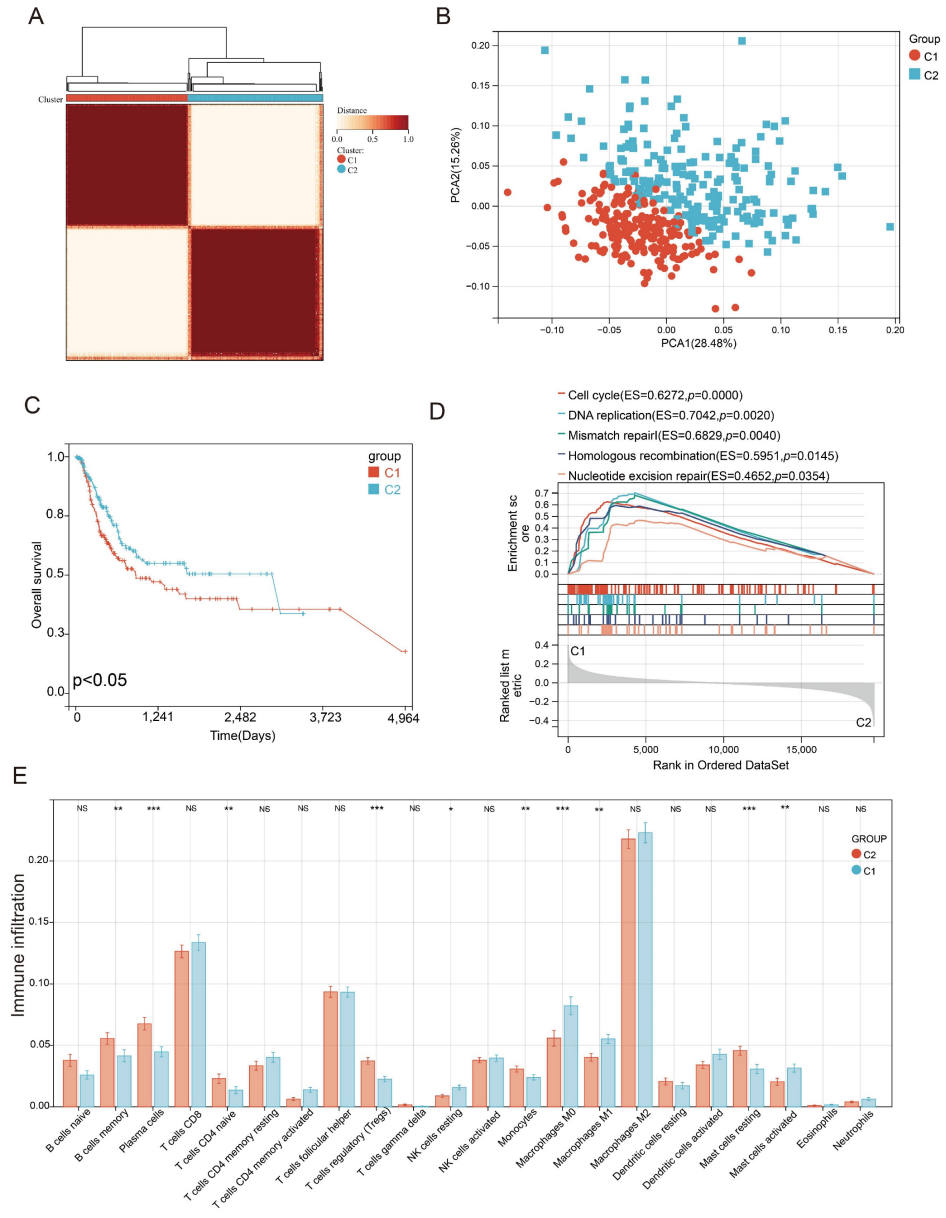
** Identification of proliferation essential gene (PEG) subtypes in the BLCA.** (A) PEG subtypes of C1 and C2 clustered by consensus matrix heatmap. (B) PCA analysis displaying a remarkable difference between the C1 and C2 subtypes. (C) Kaplan-Meier analysis, (D) gene set enrichment analysis, and (E) abundance differences of infiltrating immune cell types between the C1 and C2 subtypes. NS: *p*>0.05; *, *p*<0.05; **, *p*<0.01; ***, *p*<0.001.

Consensus clustering correlation performed on BLCA-TCGA samples based on the 201 differentially expressed PEGs yielded two distinct PEG subtypes (C1 and C2, Figure 2A), which were well distinguished as suggested by the PCA analysis (Figure 2B). We also found that BLCA patients with subtype C1 had a worse prognosis than those with the C2 subtype (Figure 2C). Genes highly expressed in subtype C1 were mainly enriched in the cell cycle, DNA replication, mismatch repair, homologous recombination, and nucleotide excision repair (Figure 2D). Furthermore, immune infiltration suggested that 10 infiltrated immune cell subtypes display great differences between the C1 and C2 subtypes (Figure 2E). Among them, Plasma cells, T cells regulatory, monocytes and mast cells resting, and immune-related B cells memory and T cells CD4 native significantly decreased in BLCA patients with the C1 subtype. In contrast, NK cells resting, macrophages M0, and inflammation-related macrophages M1 and mast cells activated markedly increased in the C1 subtype. These results suggested that anti-tumor immune responses were inhibited and the inflammatory processes were activated in the C1 subtype compared to the C2 subtype.

### Construction of a 10-PEG signature based prognostic model for BLCA patients

**Figure 3:**
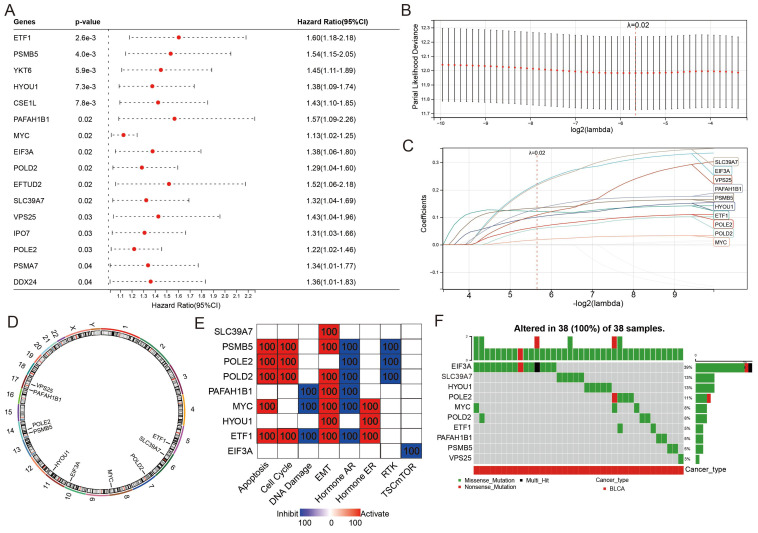
** Construction of a proliferation essential gene (PEG) signature predicting the prognosis of the BLCA.** (A) Poor prognosis associated PEGs identified by univariate Cox regression analysis in the BLCA. (B) The optimal lambda determined by partial likelihood deviation of the least absolute shrinkage and selection operator (LASSO) coefficient profiles. (C) LASSO coefficient distribution of the 10 PEGs used for signature construction. (D) The chromosomal locations of the 10 prognostic PEGs. (E) The associations between the 10 PEGs and the biological pathways involved. (F) The mutation landscape of the 10 PEGs in BLCA patients from the TCGA-BLCA dataset.

The classification of the two distinct molecular PEG subtypes implies a potential prognostic PEG biomarker, thus we established a PEG signature-based prognostic model. PEGs with prognostic values were identified and used to construct the model. OS analyses revealed that 16 PEGs out of the 198 up-regulated DEGs were significantly associated with poor prognosis in BLCA patients (Figure 3A). To select the predictive genes, a LASSO regression model was used, resulting in 10 prognostic PEGs (Figure 3B-C). Genomic feature analyses showed that the 10 PEGs are located on autosomes (Figure 3D) and are mainly involved in inhibiting hormone AR and activating the apoptosis, cell cycle and EMT pathways (Figure 3E), which are associated with the advancement and spread of cancer. As gene mutations play a crucial role in the progression of cancer, we examined the mutation patterns of the 10 PEGs in 38 BLCA patients with more than one mutated PEGs. The results showed that EIF3A had the highest mutation frequency (39%), followed by SLC39A7 (13%), HYOU1 (13%), POLE2 (11%), MYC (8%), POLD2 (8%), ETF1 (5%), PAFAH1B1 (5%), PSMB5 (5%), and VPS25 (3%) (Figure 3F). Accordingly, the prognostic PEG model based on the 10 PEGs was constructed as follows: (0.222 × EIF3A expression) + (0.121 × ETF1 expression) + (0.101 × HYOU1 expression) + (0.018 × MYC expression) + (0.131 × PAFAH1B1 expression) + (0.058 × POLD2 expression) + (0.065 × POLE2 expression) + (0.134 × PSMB5 expression) + (0.217 × SLC39A7 expression) + (0.108 × VPS25 expression).

### Differences in genomic and clinical features between patients with PEGs-low and PEGs-high subtypes

**Figure 4:**
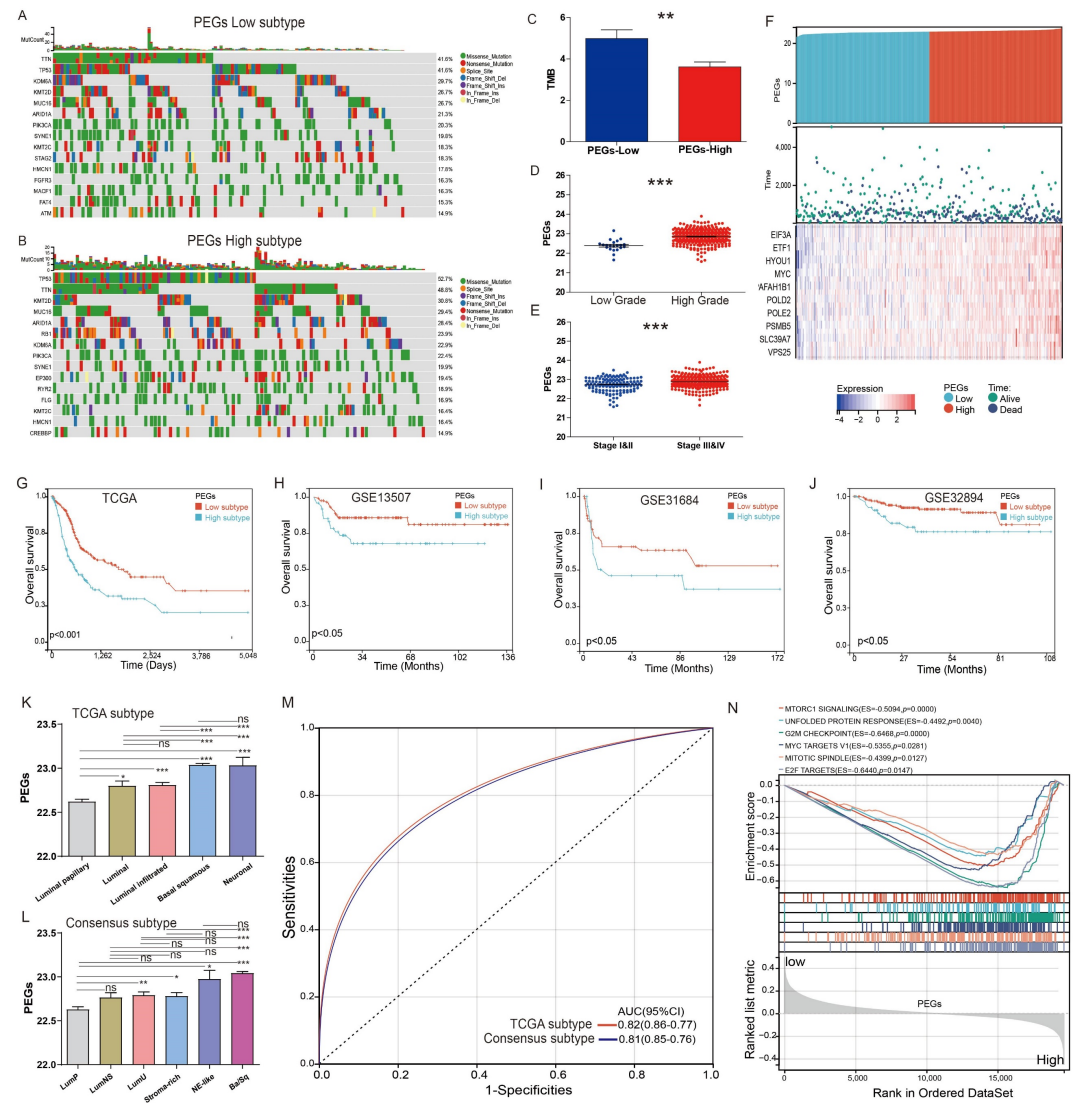
** Differences in clinicopathological characteristics and survival between BLCA patients with the proliferation essential genes (PEGs)-low and -high subtypes.** (A&B) Landscape of genomic alterations in patients with (A) PEGs-low and (B) PEGs-high subtypes. (C) Differences in tumor mutation burden (TMB) between the PEGs-low and PEGs-high subtypes. (D) Difference of the PEG signature score in BLCA patients with low and high grades. (E) Difference of the PEG signature score in BLCA patients with different stages. (F) Risk score and survival status distributions, and mRNA expression of the 10 prognostic PEGs in patients from the TCGA-BLCA dataset. (G-J) The overall survival differences between patients with PEGs-low and PEGs-high subtypes from the TCGA-BLCA, GSE13507, GSE31684 and GSE32894 datasets. (K) Differences in the PEG score between the five different molecular subtypes based on TCGA system 9. (L)Differences in the PEG score between the six molecular subtypes based on the Consensus system 10. Luminal papillary: LumP, luminal nonspecified: LumNS, luminal unstable: LumU, stroma-rich, basal/squamous: Ba/Sq, and neuroendocrine-like: NE-like. (M) ROC curves showing the accuracy of the PEG signature in prediction of the TCGA and Consensus molecular subtypes. (N) Hallmark enrichment in the PEGs-low and PEGs-high subtypes. NS: *p*>0.05; *, *p*<0.05; **, *p*<0.01; ***, *p*<0.001.

The TCGA-BLCA patients were classified into PEGs-low and PEGs-high subtypes according to the PEG model using the median score as the cutoff. We compared genomic changes between the PEGs-low and PEGs-high subtypes to explore potential molecular mechanisms. Although the constituents of the top 15 genes with the highest mutation frequency in the PEGs-low and PEGs-high subtypes were similar, almost all these genes in the PEGs-high subtype (such as TP53 gene) exhibited higher mutation rates than in the PEGs-low subtype (Figure 4A-B). Due to the production of immunogenic neoantigens, tumor mutation burden (TMB) has become a potential biomarker for immunotherapy 28, 29. Therefore, we analyzed the differences in TMB between the two subtypes, and the results showed that the PEGs-low subtype had a higher level of TMB compared to the PEGs-high subtype (Figure 4C). We also compared the relationship between the PEG signature and clinical characteristics. The PEG signature scores in high-grade BLCA were significantly higher than in low-grade BLCA patients (Figure 4D). Similarly, the PEG signature scores in BLCA patients with Stage III&IV subtypes were significantly higher than those in Stage I&II subtypes (Figure 4E). Moreover, the PEGs-high patients had higher expression of the 10 PEGs and shorter survival time or were already dead, indicating a poor prognosis of the PEGs-high patients (Figure 4F&G).

Furthermore, to confirm the robust prognosis of the signature, TCGA-BLCA patients were further categorized by various characteristics such as gender, age, and stages. Comparison of the OS differences indicated that the PEGs-high group showed a significantly lower OS than the PEGs-low group in all subgroups of male, female, age ≤ 60, age > 60, and eight disease stages (Figure S1). Consistently, three external validation datasets of GSE13507, GSE31684, and GSE32894 terminated proved that the PEGs-high subtype had a worse OS than the PEGs-low subtype (*p* <0.05) (Figure 4H-J).

### The significant correlations between the PEG signature and previously defined molecular subtypes

The TCGA molecular system comprises of five molecular classes: luminal-papillary, luminal-infiltrated, luminal, basal-squamous, and neuronal. Among them, the luminal-papillary subtype has the best prognosis, whereas the neuronal subtype has the worst prognosis 9. The Consensus molecular system consists of six molecular classes: luminal papillary (LumP), luminal nonspecified (LumNS), luminal unstable (LumU), stroma-rich, basal/squamous (Ba/Sq), and neuroendocrine-like (NE-like). In comparison to other subtypes, the LumP subtype has a better prognosis, while the NE-like and Ba/Sq subtypes have a worse prognosis 10. We examined the correlation between the PEG score and these molecular subtypes. In the TCGA molecular system, the luminal papillary with the best prognosis had the lowest PEG score (Figure 4K). Consistently, in the Consensus molecular system, the best prognostic LumP had the lowest PEG score while the worse prognostic NE-like and Ba/Sq had higher scores (Figure 4L). Regardless of the molecular systems, the majority of BLCA samples were categorized as basal squamous or luminal papillary subtypes, which most likely cover the molecular characteristics of most of BLCA patients. Other subgroups had a small-size representation, such as the neuronal subtype with only 4% of total samples in the TCGA molecular system 9 and the NE-like subtype with 2% and the stroma-rich subtype with 8% of the samples in the consensus molecular system 10. Furthermore, the ROC curves demonstrated that the PEG score can effectively predict the classical molecular subtypes, supporting by high AUC values of 0.81 for the TCGA subtype and 0.82 for the consensus subtype (Figure 4M). These findings suggest that the PEG score can effectively reflect the molecular characteristics of the majority of BLCA patients.

Additionally, the hallmark enrichment analysis results demonstrated significant enrichment of cell proliferation-related gene sets in the PEGs-high subtype, including the G2M checkpoint, MYC targets V1, E2F targets, and other signaling pathways (Figure 4N). Consequently, targeting these pathways holds a considerable therapeutic potential for patients with the PEGs-high subtype of BLCA.

### PEG signature predicts the response to chemotherapy in BLCA patients

**Figure 5:**
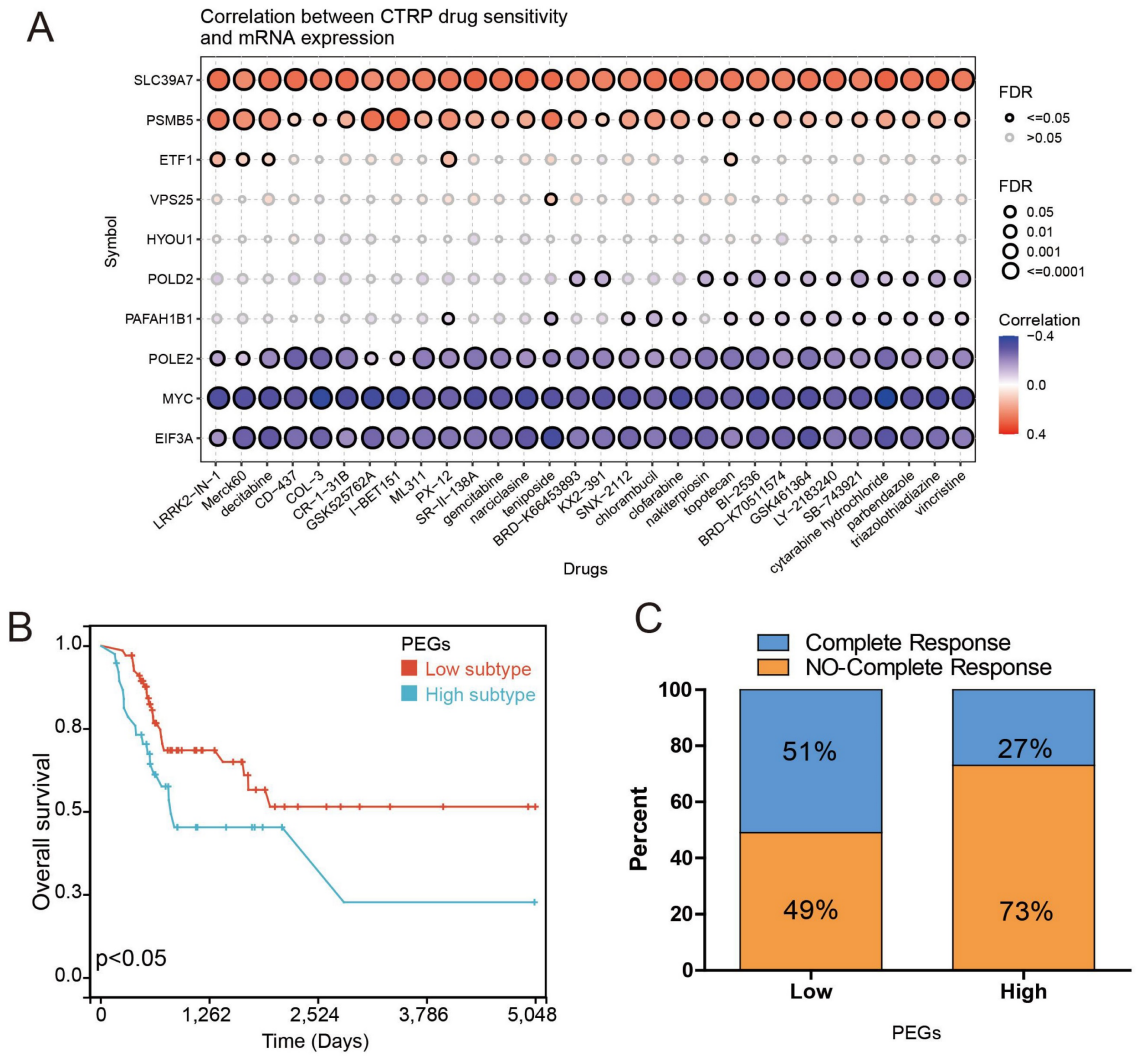
** Drug sensitivity prediction and chemotherapy response comparisons in the BLCA patients.** The correlation between GDSC drug sensitivity and gene expression of the 10 prognostic proliferation essential genes (PEGs). (B) Overall survival difference between the PEGs-low and PEGs-high BLCA patients who received chemotherapy. (C) Comparison of chemotherapy responses in BLCA patients with PEGs-low and PEGs-high subtypes.

Chemotherapy can improve the prognosis of BLCA patients compared to surgery alone. However, drug resistance remains a major obstacle to chemotherapy response. We investigated the correlation between drug sensitivity and expression of the PEGs based on the CTRP database to estimate the chemotherapy response. The results showed that the gene expression of SLC39A7 and PSMB5 were positively correlated with the IC50 of anticancer drugs, while the expression of POLE2, MYC, and EIF3A were opposite (Figure 5A). These results can be used to guide the development of chemotherapy regimens. Then, we investigated the correlation between gene expression of the PEGs and chemotherapy response in 109 BLCA patients who had received chemotherapy treatment based on the TCGA database. The results showed that compared to the PEGs-high BLCA patients who received chemotherapy, the PEGs-low patients who received chemotherapy experienced more benefits in OS (Figure 5B). It is noteworthy that in the PEGs-low subtype, 51% of BLCA patients achieved complete remission after chemotherapy treatment. In contrast, only 27% of BLCA patients achieved complete remission after chemotherapy treatment in the PEGs-high subtype (Figure 5C). These findings suggest that BLCA patients with low PEG signature may be more sensitive to chemotherapy treatment and the PEG signature will benefit the prediction of the response to chemotherapy treatment in BLCA patients.

### POLE2 expression increases in BLCA tissues and is associated with poor survival in patients

**Figure 6:**
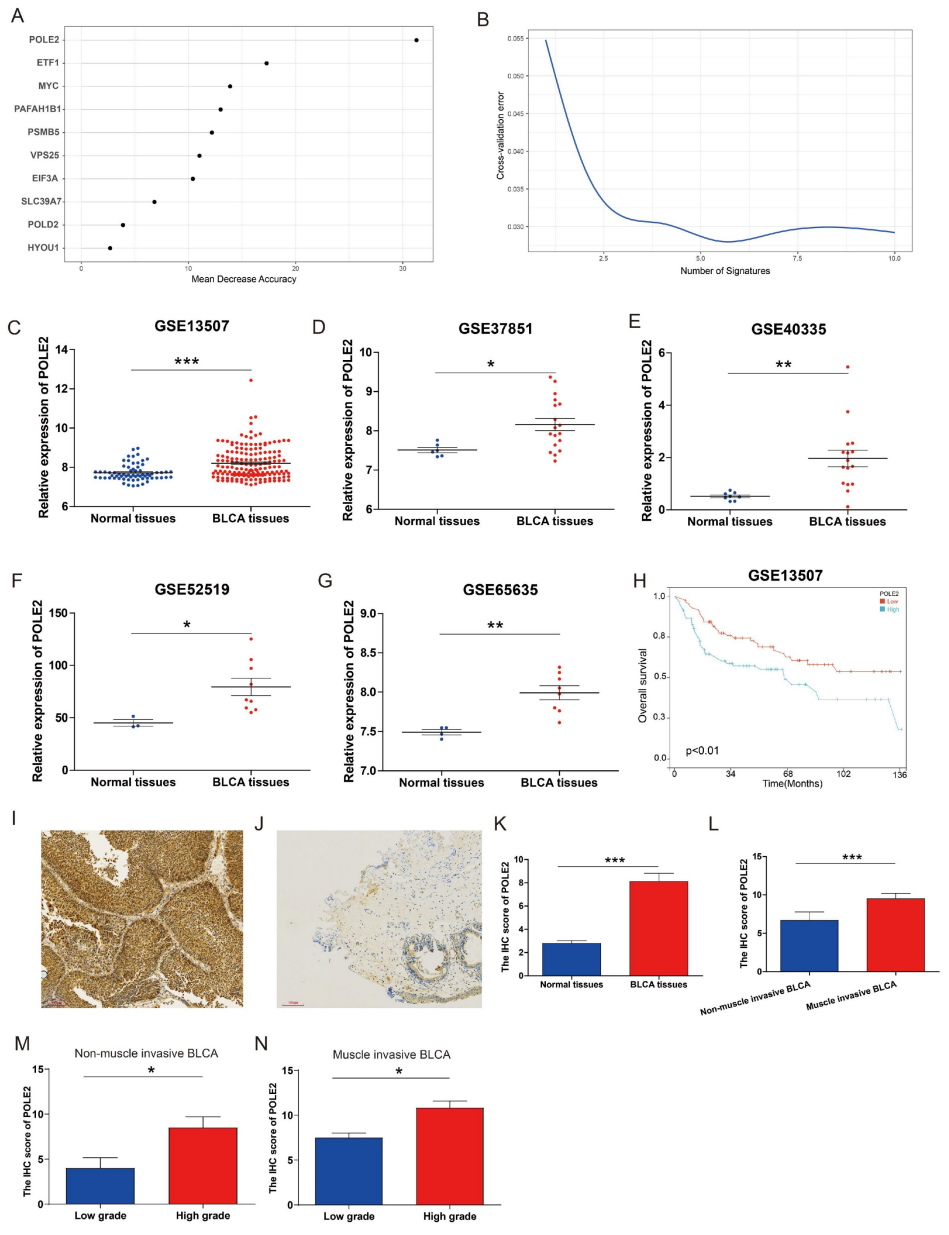
** POLE2 expression in the BLCA and its relationship with overall survival.** (A&B) Random forest displaying the most important proliferation essential gene (PEG) — POLE2 among the 10 prognostic PEGs. (C-G) Comparisons of POLE2 expression between the BLCA and normal tissues from the datasets (C) GSE13507, (D) GSE37851, (E) GSE40335, (F) GSE52519 and (G) GSE65635. (H) Overall survival difference in BLCA patients with high- and low-POLE2 expression from the GSE13507 dataset. (I&J) Representative immunohistochemistry (IHC) images of POLE2 protein in (I) the BLCA tissues and (J) paired normal urothelial tissues. (K) Histogram of the IHC score revealing markedly higher protein expression of POLE2 in BLCA tissues than in normal tissues. (L) An enhanced protein level of POLE2 in the muscle-invasive BLCA compared to the non-muscle invasive BLCA suggested by the IHC assay. (M) Comparison of POLE2 protein levels between the high-grade non-muscle invasive BLCA and the low-grade non-muscle invasive using the IHC assay. (N) Comparison of POLE2 protein levels between the high-grade muscle invasive BLCA and the low-grade muscle invasive using the IHC assay. *, *p*<0.05; **, *p*<0.01; ***, *p*<0.001.

To explore the molecular regulation mechanism of PEGs, we performed forest analysis to target the most important gene as a representative PEG that was used for further investigation. Our analysis revealed that POLE2 was the most significant gene among the 10 PEGs (Figure 6A&B). To further investigate the molecular characteristics of POLE2 in BLCA, we detected its transcript expression in five independent GEO datasets (GSE13507, GSE37851, GSE40335, GSE52519, and GSE65635) and protein expression in clinical samples using IHC assay. Results suggested that POLE2 was significantly higher expressed in BLCA tissues than in the normal tissues (Figure 6C-G). Moreover, patients with high POLE2 gene expression have worse OS than those with low POLE2 gene expression in the GSE13507 dataset (Figure 6H). IHC staining results consistently demonstrated that POLE2 in BLCA tissues had higher protein expression than in normal urothelial tissues (Figure 6I-K). Meanwhile, the protein expression of POLE2 in muscle-invasive BLCA was dramatically higher than the expression in non-muscle invasive BLCA (Figure 6L). Further analysis revealed that the expression of the POLE2 protein was higher in high-grade muscle invasive BLCA than in the low-grade muscle invasive BLCA, and also higher in the high-grade non-muscle invasive BLCA c than the low-grade non-muscle invasive BLCA (Figure 6M&N), suggesting a potential association between POLE2 and the progression of BLCA.

### Knockdown of POLE2 inhibits BLCA cell stemness, proliferation, invasion, migration and chemoresistance

**Figure 7:**
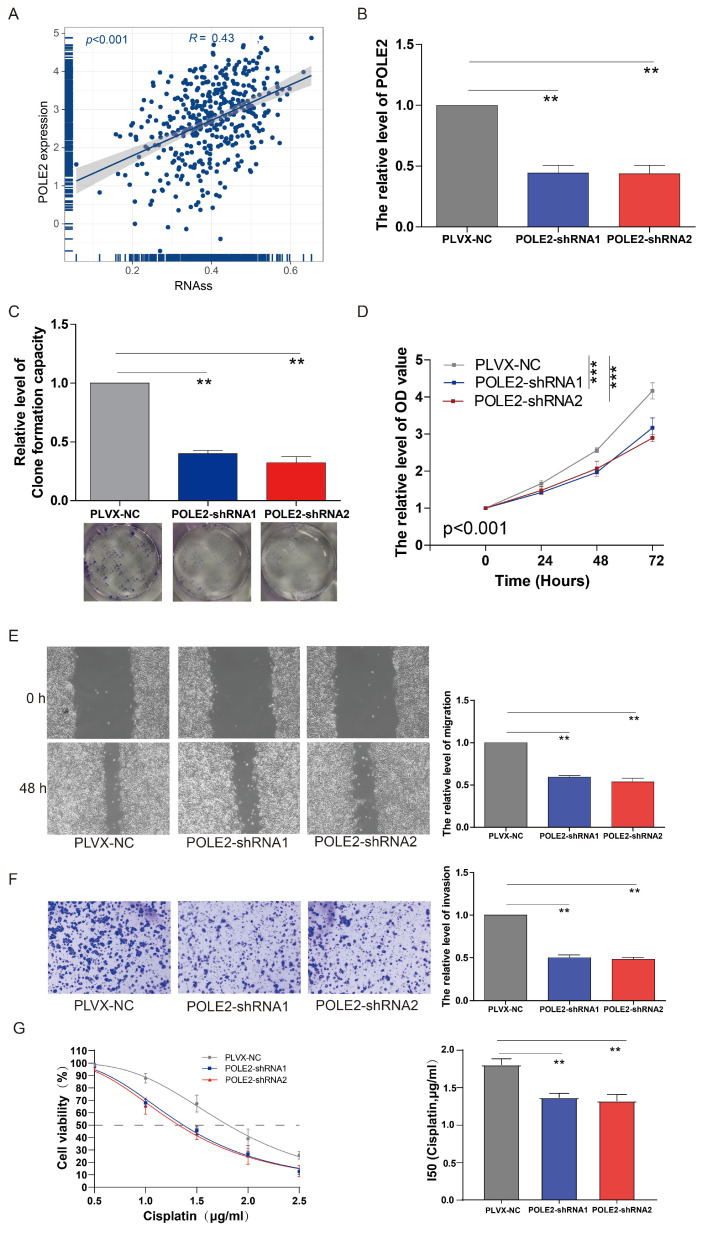
** The effects of POLE2 knockdown on the BLCA cell stemness, proliferation, invasion and migration.** (A) The relationship between the POLE2 expression and RNA stemness score (RNAss) in the BLCA. (B) POLE2 expression in the T24 cells transfected with POLE2-short hairpin RNAs (POLE2-shRNAs, including POLE2-shRNA1 and POLE2-shRNA2). (C-F) Effects of POLE2 knockdown on (C) T24 cell stemness assessed by the clonogenic assay, (D) T24 cell growth determined by the CCK-8 assay, (E) T24 cell migration distance after 48 hours transfection determined by the scratch assay, and (F) the T24 cell invasion capability. (G) Effects of POLE2 knockdown on cisplatin chemotherapy resistance. **, *p*<0.01.

Our findings suggested that the POLE2 gene was crucial for cell proliferation, but its exact function in regulating cancer cell stemness is yet unknown in BLCA. According to the correlation analysis, we found that POLE gene expression was positively correlated with the RNAss in BLCA (*R*= 0.43, *p* < 0.001, Figure 7A). A clonogenic assay was conducted for assessing the effects of POLE2 on T24 cell stemness. Successful transfection of two POLE2-targeting short hairpin RNAs (shRNAs, POLE2-shRNA1 and POLE2-shRNA2) into T24 cells was demonstrated by the markedly reduced expression of PLOE2 after POLE2 shRNA as quantified by qRT-PCR (Figure 7B).

Cell clone formation analysis revealed that POLE2 knockdown significantly decreased the capacity of cell clone formation in POLE2-shRNA1 and POLE2-shRNA2 (Figure 7C). In addition, POLE2-shRNA1 and POLE2-shRNA2 cells were employed to detect changes in cell proliferation, invasion and immigration abilities. POLE2 inhibition greatly hindered the proliferation ability, as evidenced by the significantly decreased cell viability OD values (*p* < 0.001) of POLE2-shRNA1 and POLE2-shRNA2 cells compared to the control empty plasmid pLVX-NC (Figure 7D). Additionally, the POLE2 suppression significantly inhibited both migration and invasion of T24 cells (Figure 7E&F). Finally, we assessed the impact of POLE2 expression on the resistance to cisplatin, a commonly used chemotherapy drug for bladder cancer. The results indicated that knocking down POLE2 can significantly lower the IC50 of cisplatin, suggesting that POLE2 plays an important role in the resistance to cisplatin chemotherapy (Figure 7G).

## Discussion

BLCA has a high propensity for multiple recurrences and disease progression. It is also the most expensive neoplastic disease to treat on a per-patient basis which causes a serve burden for healthcare systems 30. Inadequate prognostication and prediction of disease progress persist even though most cases can be cured by a combined modality strategy of surgery, chemotherapy, and radiation 31. Molecular biomarkers and new therapeutic targets for individualized diagnosis and prognosis of BLCA aid in stratification and precision medicine for BLCA patients 32, 33, 34. Excepting for existing BLCA molecular subtype systems 9, 10, new molecular classification systems are required for BLCA precision medicine due to its lower inspection cycle and lower cost. Oncogenes and tumor suppressor genes that are crucial for the development and survival of cancer cells are known as selective essential genes. They confer properties on cancer cells differing from normal cell and provide a new way for selecting therapeutic targets. The identification of PEGs in BLCA will bring new insights into understanding tumorigenesis and therapeutic strategies.

Genome-wide CRISPR-Cas9 loss-of-function screening is a wildly useful tool to explore gene function in tumor cells 35, 36, 37. Users can assess whether genes are essential for tumor cells using the Depmap database, which integrates CRISPR-Cas9 screen datasets across 342 cancer cell lines and applied CERES to the dataset 24. Taking advantage of the DepMap project, we identified 699 genes essential for BLCA cell proliferation based on CERES scores. Differential expression analysis of the TCGA-BLCA dataset indicated that the majority of the DEGs (189/201) were up-regulated in BLCA tissues and mainly take part in the cell cycle, suggesting their critical involvement in BLCA cell proliferation. Two subtypes (C1 and C2) were distinguished based on the clustering of the 201 differentially expressed PEGs. GSEA and immune infiltration results suggested that the dysregulated cell cycle pathway, inhibited anti-tumor immune responses and the enhanced inflammatory process probably contribute to the poor prognosis of patients with the C1 subtype compared to those with the C2 subtype. Meanwhile, 10 dysregulated PEGs with OS prognostic values were screened and used to construct a PEG signature based prognostic model that classifies patients into PEGs-low and PEGs-high subtypes. The lower OS of patients with the PEGs-high subtype compared to those with the PEGs-low subtype in many datasets (the TCGA-BLCA dataset, validated GEO datasets and sub-datasets categized by male, female, young and older, disease grades and disease stages) demonstrated that the gene signature has a good performance in prediction of OS. In addition, the PEG signature has the ability to accurately predict classical molecular subtypes. We also found that compared to the PEGs-high subtype, patients in the PEGs-low subtype had a better prognosis after chemotherapy treatment. The proportion of BLCA patients in the PEGs-low subtype who achieved complete response after chemotherapy was higher than in the PEGs-high subtype. These results indicate that the PEG signature is a useful predictive tool for chemotherapy treatment in BLCA.

Based on the random forest analysis of the 10 PEGs, we discovered that POLE2 was the most significant gene differentiating bladder cancer tissues and adjacent normal tissues, implying that it plays an extremely important role in BLCA progression. The markedly increased expression of POLE2 in BLCA tissues from five GEO datasets suggests that it is a poor prognostic factor of BLCA. Classical DNA polymerases are primarily categorized into five types: Pol α, Pol β, Polγ, Pol δ and Pol ε. These polymerases are involved in DNA replication and repair 38 and have been linked to the development of cancer 39, 40.

Polε is involved in the DNA synthesis of the leading strand, and its subunits comprise POLE1, POLE2, POLE3 and POLE4 38. The C-terminal half of POLE1 is required for interaction with POLE2. While the interaction between POLE1 and POLE2 is essential for supporting DNA synthesis. POLE3 and POLE4 aid the processivity of DNA replication. POLE2 takes part in DNA replication, base excision repair, and nucleotide excision repair 41, 42. Thus, it is not supervising that POLE2 is associated with cancer progression as its essential role in DNA replication.

The gene mutations of POLE2 in breast cancer, colorectal cancer, and endometrial cancer are tightly associated with the occurrence, development, and prognosis of cancers 43, 44, 45, 46. It was reported that the POLE2 gene is increased in mantle cell lymphoma, esophageal squamous cell, glioblastoma and lung cancer 47, 48, 49, 50. In squamous cell lung cancer, the up-regulated expression of the POLE2 gene was inversely correlated with survival and immune infiltration 48. High expression of the POLE2 gene is also associated with poor prognosis in renal cell carcinoma 51, 52. Similarly, our results demonstrated that POLE2 is up-regulated in BLCA tissues at both the transcriptional and protein levels and is associated with a poor prognosis. The POLE2 knockdown cells exhibit inhibited the ability of cell clone formation, proliferation, invasion and immigration, which is consistent with findings in lung adenocarcinoma cells 53, indicating that POLE2 is a bladder tumor promotor. To be noted, as POLE2 is a DNA replication related gene that plays commonly essential DNA synthesis function in all cell lines and has a median score below -2 in Depmap, POLE2 might also be a tumor promotor and therapeutic target in other cancers which needs further investigation.

The inhibited cell ability of POLE2 knockdown as well as the positive correlation between POLE2 expression and cell stemness demonstrate its vital role in BLCA oncogenesis. However, the molecular mechanisms of POLE2 on BLCA tumorigenesis are poorly understood, despite several studies that have recently shown different molecular regulation mechanisms of POLE2 in other cancers. POLE2 knockdown in lung cancer activates cellular iron-dependent ferroptosis by increasing the production of lipid ROS, MDA and iron content in cells through modulation of P53 expression or PI3K/AKT signaling 54. In glioblastoma, POLE2 knockdown suppresses cell proliferation and metastasis by controlling AURKA (Aurora kinase A) to promote ubiquitination and reduce the stability of the tumor-promoting factor FOXM1 (forkhead transcription factor) 50. Su et al. discovered that the knockdown of POLE2 in renal cell carcinoma causes the cell cycle to stop at the S phase and increase cell apoptosis via AKT/mTOR signaling 51, whereas Zhang et al. found that POLE2 knockdown attenuates cell proliferation and migration by reducing the expression of its downstream gene STC1 (Stanniocalcin 1) gene, which is a tumor promoter 52. Further clarification of the underlying mechanisms of POLE2 on BLCA progression is of extreme significance.

## Conclusion

In conclusion, we developed a 10-PEG signature-based prognostic model for BLCA patients. The PEG signature provides a potential way to stratify BLCA patients and predict who will benefit more if receiving chemotherapy treatments. POLE2 is the most predictive PEG which is up-regulated in BLCA tissues and associated with BLCA progression.

## Supplementary Material

Supplementary figure and table.
