# Supplementary material for: Prognostic and chemotherapeutic response prediction by proliferation essential gene signature: Investigating POLE2 in bladder cancer progression and cisplatin resistance
Source: J Cancer. 2024 Feb 4;15(6):1734–49. doi: 10.7150/jca.93023 (PMC10869977; doi:10.7150/jca.93023)
Supplement: Supplementary file 1 — Supplementary figure and table. [file jcav15p1734s1.pdf]

Figure S1. Survival analyses of BLCA patients with PEGs-high and PEGs-low subtypes from various subgroups. Comparisons of the overall survival differences between the PEGs-high and the PEGs-low groups in the BLCA patient subgroups categorized by (A) male and (B) female, (C) age ≤60 years, (D) age >60 years, (E) grade=1-2, (F) grade=3-4, (G) stage T=1-2, (H) stage T=3-4, (I) stage M=0, (J) stage M=1&X, (K) stage N=0, and (L) stage N=1&X.

Figure S1

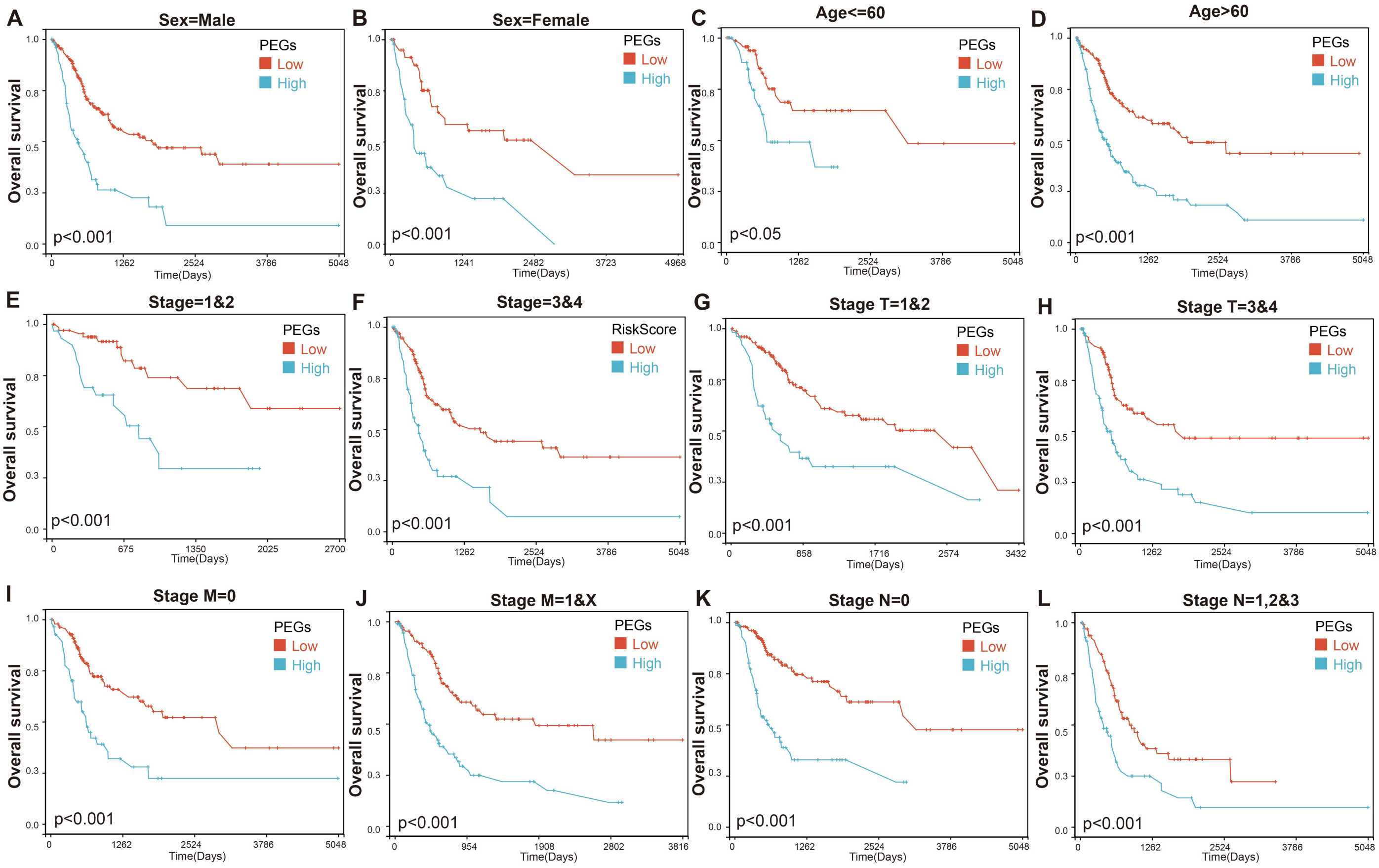
A
Sex=Male
B
Sex=Female
C
Age<=60
D
Age>60
E
Stage=1&2
F
Stage=3&4
G
Stage T=1&2
H
Stage T=3&4
I
Stage M=0
J
Stage M=1&X
K
Stage N=0
L
Stage N=1,2&3
PEGs
RiskScore
Low
High
Overall survival
Time(Days)
p<0.001
p<0.05

Table S1. Potential proliferation-essential genes for BLCA identified base on Depmep.

NO.	Gene symbol
1	AARS
2	ABCE1
3	ABCF1
4	ACTL6A
5	ACTR2
6	AHCTF1
7	AKIRIN2
8	ALG11
9	ALG1L
10	ALG2
11	ANAPC1
12	ANAPC10
13	ANAPC11
14	ANAPC2
15	ANAPC4
16	ANAPC5
17	ANKLE2
18	AQR
19	ARCN1
20	ARIH1
21	ARL2
22	ARMC7
23	ATP2A2
24	ATP5MF
25	ATP6V0B
26	ATP6V0C
27	ATP6V1A
28	ATP6V1B2
29	ATP6V1E1
30	ATP6V1F
31	ATP6V1G1
32	AURKA
33	AURKB
34	BANF1
35	BIRC5
36	BRF1
37	BRF2
38	BRIX1
39	BUB1B
40	BUB3
41	BUD23
42	BUD31
43	BYSL
44	C1orf109
45	CACTIN
46	CARS
47	CCDC84
48	CCNA2
49	CCNK

50	CCT2
51	CCT3
52	CCT4
53	CCT5
54	CCT6A
55	CCT7
56	CCT8
57	CDC123
58	CDC16
59	CDC20
60	CDC23
61	CDC27
62	CDC37
63	CDC45
64	CDC5L
65	CDC6
66	CDC7
67	CDC73
68	CDCA8
69	CDK1
70	CDK7
71	CDK9
72	CDT1
73	CENPK
74	CENPN
75	CENPW
76	CFAP298
77	CHAF1B
78	CHEK1
79	CHMP2A
80	CHMP6
81	CIAO1
82	CIAO2B
83	CIAO3
84	CKAP5
85	CLNS1A
86	CLP1
87	CLTC
88	CNIH4
89	CNOT3
90	COPA
91	COPB1
92	COPB2
93	COPE
94	COPS5
95	COPS6
96	COPZ1
97	CPSF1
98	CPSF2
99	CPSF3
100	CPSF4
101	CRCP
102	CRNKL1

103	CSE1L
104	CTCF
105	CTDP1
106	CWC22
107	DAD1
108	DARS
109	DBR1
110	DCTN5
111	DDB1
112	DDX10
113	DDX18
114	DDX20
115	DDX21
116	DDX24
117	DDX41
118	DDX42
119	DDX46
120	DDX47
121	DDX49
122	DDX54
123	DDX55
124	DDX56
125	DHDDS
126	DHX15
127	DHX16
128	DHX33
129	DHX37
130	DHX8
131	DKC1
132	DNAJC17
133	DNAJC8
134	DNM2
135	DONSON
136	DPAGT1
137	DTL
138	DTYMK
139	DUT
140	DYNC1H1
141	DYNC1I2
142	DYNLRB1
143	ECD
144	EEF1A1
145	EEF1G
146	EEF2
147	EEF2KMT
148	EFTUD2
149	EIF1AX
150	EIF2B2
151	EIF2B3
152	EIF2B4
153	EIF2B5
154	EIF2S1
155	EIF2S2

156	EIF2S3
157	EIF3A
158	EIF3B
159	EIF3D
160	EIF3E
161	EIF3F
162	EIF3G
163	EIF3I
164	EIF4A1
165	EIF4A3
166	EIF4E
167	EIF5
168	EIF6
169	ELL
170	ELOB
171	EPRS
172	ERH
173	ESPL1
174	ESS2
175	ETF1
176	EXOSC4
177	EXOSC6
178	EXOSC7
179	FARSA
180	FARSB
181	FAU
182	FBL
183	FCF1
184	FNTA
185	GARS
186	GEMIN5
187	GGTLC2
188	GINS1
189	GINS2
190	GINS3
191	GINS4
192	GNL3
193	GPN1
194	GPN2
195	GPN3
196	GPS1
197	GRPEL1
198	GSPT1
199	GTF2A2
200	GTF2B
201	GTF2E1
202	GTF2E2
203	GTF2F2
204	GTPBP4
205	GUK1
206	HARS
207	HAUS1
208	HAUS5

209	HAUS6
210	HAUS7
211	HAUS8
212	HCFC1
213	HEATR1
214	HINFP
215	HIST1H2AK
216	HIST1H2BC
217	HIST1H2BD
218	HIST1H2BE
219	HIST1H2BJ
220	HIST1H2BN
221	HIST2H3D
222	HMGCS1
223	HNRNPC
224	HNRNPK
225	HNRNPL
226	HNRNPM
227	HSPA5
228	HSPA9
229	HSPD1
230	HSPE1
231	HYOU1
232	IARS
233	IFITM3
234	IGBP1
235	IK
236	ILF2
237	IMP3
238	INCENP
239	INTS11
240	INTS3
241	INTS4
242	INTS9
243	IPO7
244	ISCU
245	ISY1
246	KARS
247	KAT8
248	KIF11
249	KIF23
250	KIN
251	KPNB1
252	KRR1
253	KRT8
254	LARS
255	LAS1L
256	LCE5A
257	LONP1
258	LRR1
259	LSG1
260	LSM11
261	LSM2

262	LSM3
263	LSM4
264	LSM5
265	LSM6
266	LSM7
267	LSM8
268	LTO1
269	LUC7L3
270	MAD2L1
271	MAK16
272	MARS
273	MASTL
274	MCM2
275	MCM3
276	MCM4
277	MCM5
278	MCM6
279	MCM7
280	MDN1
281	MED11
282	MED14
283	MED22
284	MED28
285	MED30
286	MED6
287	MED8
288	MEPCE
289	MFAP1
290	MIS18A
291	MMS22L
292	MPHOSPH10
293	MTBP
294	MTREX
295	MYC
296	MZT1
297	NAA10
298	NAA50
299	NACA
300	NAPA
301	NARS
302	NCAPG
303	NCBP1
304	NCBP2
305	NDC80
306	NEDD1
307	NEDD8
308	NFS1
309	NIFK
310	NIP7
311	NLE1
312	NMD3
313	NOB1
314	NOC4L

315	NOL10
316	NOL6
317	NOP16
318	NOP2
319	NOP56
320	NOP58
321	NPLOC4
322	NRF1
323	NSA2
324	NSF
325	NUDT21
326	NUF2
327	NUP133
328	NUP160
329	NUP214
330	NUP85
331	NUP88
332	NUP93
333	NUS1
334	NUTF2
335	NVL
336	NXF1
337	OGT
338	OIP5
339	ORC1
340	ORC6
341	PABPN1
342	PAFAH1B1
343	PAM16
344	PCID2
345	PCNA
346	PDCD11
347	PDCD2
348	PDRG1
349	PELP1
350	PFDN2
351	PFDN6
352	PHAX
353	PHB
354	PHB2
355	PHF5A
356	PLK1
357	PMF1
358	PMPCB
359	PNN
360	POLA2
361	POLD1
362	POLD2
363	POLD3
364	POLE
365	POLE2
366	POLR1A
367	POLR1B

368	POLR1C
369	POLR2B
370	POLR2C
371	POLR2D
372	POLR2E
373	POLR2F
374	POLR2G
375	POLR2H
376	POLR2I
377	POLR2L
378	POLR3A
379	POLR3B
380	POLR3C
381	POLR3F
382	POLR3H
383	POLR3K
384	POP5
385	PPAN
386	PPWD1
387	PRC1
388	PREB
389	PRELID1
390	PRELID3B
391	PRIM1
392	PRMT5
393	PRPF19
394	PRPF31
395	PRPF38A
396	PRPF38B
397	PRPF6
398	PRPF8
399	PSMA1
400	PSMA2
401	PSMA3
402	PSMA4
403	PSMA5
404	PSMA6
405	PSMA7
406	PSMB1
407	PSMB2
408	PSMB3
409	PSMB4
410	PSMB5
411	PSMB7
412	PSMC1
413	PSMC2
414	PSMC3
415	PSMC4
416	PSMC5
417	PSMC6
418	PSMD1
419	PSMD11
420	PSMD12

421	PSMD14
422	PSMD2
423	PSMD3
424	PSMD4
425	PSMD6
426	PSMD7
427	PSMD8
428	PSMG3
429	PSMG4
430	PUF60
431	PWP2
432	QARS
433	RABGGTA
434	RABGGTB
435	RACK1
436	RAD21
437	RAD51
438	RAE1
439	RAN
440	RANGAP1
441	RBBP4
442	RBBP6
443	RBM14
444	RBM17
445	RBM22
446	RBM25
447	RBM39
448	RBM8A
449	RBMX
450	RBX1
451	RCC1
452	RFC2
453	RFC3
454	RFC5
455	RIOK2
456	RNGTT
457	RNMT
458	RNPC3
459	RPA1
460	RPA2
461	RPA3
462	RPAIN
463	RPAP1
464	RPAP2
465	RPL10A
466	RPL11
467	RPL12
468	RPL13
469	RPL13A
470	RPL14
471	RPL15
472	RPL18
473	RPL18A

474	RPL19
475	RPL21
476	RPL23
477	RPL23A
478	RPL24
479	RPL26
480	RPL27
481	RPL27A
482	RPL3
483	RPL30
484	RPL31
485	RPL32
486	RPL35
487	RPL36
488	RPL37
489	RPL37A
490	RPL38
491	RPL4
492	RPL5
493	RPL6
494	RPL7
495	RPL8
496	RPLP0
497	RPLP1
498	RPLP2
499	RPP21
500	RPP30
501	RPP40
502	RPS10
503	RPS11
504	RPS12
505	RPS13
506	RPS15
507	RPS15A
508	RPS16
509	RPS18
510	RPS19
511	RPS2
512	RPS20
513	RPS21
514	RPS23
515	RPS24
516	RPS25
517	RPS27A
518	RPS29
519	RPS3
520	RPS4X
521	RPS5
522	RPS6
523	RPS7
524	RPS8
525	RPS9
526	RPSA

527	RRM1
528	RRM2
529	RRN3
530	RRP12
531	RSL1D1
532	RTF2
533	RUVBL1
534	RUVBL2
535	SACM1L
536	SAP18
537	SAP30BP
538	SARS
539	SART3
540	SBDS
541	SBNO1
542	SCFD1
543	SDAD1
544	SDE2
545	SEC13
546	SEC61A1
547	SEC61G
548	SF1
549	SF3A1
550	SF3A2
551	SF3A3
552	SF3B1
553	SF3B2
554	SF3B3
555	SF3B4
556	SF3B5
557	SF3B6
558	SFPQ
559	SKP1
560	SLC39A7
561	SLU7
562	SMC1A
563	SMC2
564	SMC3
565	SMC4
566	SMG1
567	SMG5
568	SMR3B
569	SMU1
570	SNAPC1
571	SNAPC2
572	SNAPC3
573	SNAPC4
574	SNAPC5
575	SNRNP200
576	SNRNP25
577	SNRNP27
578	SNRNP35
579	SNRNP70

580	SNRPA1
581	SNRPB
582	SNRPC
583	SNRPD1
584	SNRPD2
585	SNRPD3
586	SNRPF
587	SNU13
588	SNW1
589	SOD1
590	SPC24
591	SPC25
592	SPCS2
593	SPDL1
594	SPOUT1
595	SRBD1
596	SRP19
597	SRP54
598	SRP72
599	SRP9
600	SRSF1
601	SRSF2
602	SRSF3
603	SRSF7
604	SS18L2
605	SSRP1
606	SSU72
607	STX5
608	SUPT16H
609	SUPT5H
610	SUPT6H
611	SYMPK
612	TAF1C
613	TAF6
614	TANGO6
615	TARS
616	TCP1
617	THOC2
618	THOC3
619	THOC5
620	TICRR
621	TIGD1
622	TIMELESS
623	TIMM10
624	TINF2
625	TNPO3
626	TOMM22
627	TOMM40
628	TONSL
629	TOP2A
630	TOPBP1
631	TRAPPC11
632	TRAPPC3

633	TRAPPC4
634	TRAPPC5
635	TRAPPC8
636	TRMT112
637	TRRAP
638	TSR1
639	TSR2
640	TTC27
641	TUBB
642	TUBG1
643	TUBGCP2
644	TUBGCP3
645	TUT1
646	TWISTNB
647	TXNL4A
648	U2AF2
649	U2SURP
650	UBA1
651	UBA2
652	UBA52
653	UBE2I
654	UBL5
655	UBTF
656	UFD1
657	UPF1
658	UPF2
659	URI1
660	USP36
661	USP39
662	USP5
663	USPL1
664	UTP15
665	UTP20
666	UTP4
667	VARS
668	VCP
669	VIRMA
670	VPS25
671	VPS28
672	WARS
673	WDR12
674	WDR3
675	WDR33
676	WDR43
677	WDR46
678	WDR5
679	WDR70
680	WDR74
681	WDR75
682	WDR77
683	WDR82
684	WEE1
685	XAB2

686	XPO1
687	XRCC6
688	YAE1
689	YARS
690	YJU2
691	YKT6
692	ZMAT2
693	ZMAT5
694	ZNF131
695	ZNF207
696	ZNF830
697	ZNHIT2
698	ZPR1
699	ZRSR2

Table S2. Differentially expressed genes between between BLCA tissues and normal tissues from the TCGA-BLCA dataset .

Tag	logFC	AveExpr	P.Value	adj.P.Val
CDK1	3.2851315	18.14003776	9.50E-38	6.41E-35
CDCA8	3.1800104	17.94803913	9.54E-33	3.22E-30
AURKB	3.6434062	18.12261699	1.94E-32	4.35E-30
CDT1	3.1534322	17.66552075	8.81E-31	1.49E-28
CDC45	3.2444045	17.14483306	4.16E-30	4.87E-28
BIRC5	3.4256901	18.35472875	4.41E-30	4.87E-28
ORC6	2.9508063	16.13779086	5.06E-30	4.87E-28
CDC20	3.537109	19.47418045	4.83E-29	4.07E-27
NCAPG	3.2122239	16.49306968	6.57E-29	4.77E-27
BUB1B	3.1092216	16.64823752	7.08E-29	4.77E-27
TOP2A	3.309628	18.92086679	8.03E-29	4.92E-27
NUF2	3.389908	16.90119893	1.96E-28	1.10E-26
RAD51	2.5477371	16.65917089	3.86E-28	2.00E-26
AURKA	2.7587484	17.76956716	6.44E-28	3.10E-26
CDC6	2.7044578	17.18478783	2.31E-27	1.04E-25
GINS1	2.6267405	17.0073885	3.35E-27	1.41E-25
DTL	2.7828968	16.2352768	7.30E-26	2.90E-24
SPC25	2.8694875	16.49118003	1.55E-25	5.80E-24
PLK1	2.9284827	17.62967233	2.27E-25	8.06E-24
CCNA2	2.7131682	17.9148757	5.04E-25	1.70E-23
RCC1	1.6061363	19.02941874	1.08E-24	3.45E-23
SPC24	2.6548324	17.10334111	2.40E-24	7.36E-23
CENPK	2.6208883	15.4541252	2.58E-24	7.55E-23
TONSL	1.8861836	16.86658182	5.56E-23	1.56E-21
RRM2	3.0264648	18.34414418	7.59E-23	2.05E-21
OIP5	2.6074245	16.05914302	3.35E-22	8.67E-21
KIF23	2.5123107	16.63859088	3.60E-22	8.98E-21
ESPL1	2.7233592	15.91017879	2.07E-21	4.98E-20
KIF11	2.290178	17.37431881	3.23E-21	7.52E-20
GINS2	2.2844708	17.08394517	5.52E-21	1.24E-19
ORC1	2.4983771	16.01015012	8.80E-21	1.91E-19
PSMG3	1.524487	19.25556939	5.49E-20	1.16E-18
NDC80	2.4733579	17.00159261	5.96E-20	1.22E-18
TIMELESS	1.6006078	17.83590446	6.59E-20	1.31E-18
POLR2H	1.1850577	19.01050958	3.26E-19	6.27E-18
PRC1	2.16394	17.65412987	9.66E-19	1.81E-17
POLD1	1.4309861	17.644242	4.12E-18	7.50E-17
POLE2	1.8337222	15.62816527	6.40E-18	1.14E-16
NUP85	0.9768826	18.12834716	7.24E-18	1.25E-16
CHEK1	1.6760577	16.23714317	5.31E-17	8.96E-16
MCM2	1.9677194	18.48993673	2.58E-16	4.24E-15
ACTL6A	1.1341082	18.76904283	3.13E-16	5.02E-15
TICRR	1.9067218	14.86829799	3.52E-16	5.52E-15
SNRPA1	1.0070074	18.0830543	8.21E-16	1.26E-14
SBDS	-1.024173	19.84669941	1.05E-15	1.57E-14
EFTUD2	0.8172456	18.33580691	1.41E-15	2.07E-14
CDC7	1.7351336	16.38003816	1.72E-15	2.47E-14
MAD2L1	1.9329323	16.61025287	3.57E-15	5.01E-14
SEC61A1	0.7781864	21.1519898	4.90E-15	6.74E-14
MTBP	1.5930034	14.29205403	5.12E-15	6.90E-14
MCM4	1.5497308	18.87941336	6.82E-15	9.01E-14
MIS18A	1.2939734	18.08906003	1.04E-14	1.34E-13
PCNA	1.4506096	21.20446437	1.78E-14	2.26E-13
LSM4	1.0449991	19.84568103	2.30E-14	2.87E-13

DTYMK	1.3030695	18.43318541	2.69E-14	3.30E-13
RFC5	1.1140138	17.48100957	4.12E-14	4.96E-13
SNRPB	1.1495579	22.2701266	4.78E-14	5.65E-13
CHAF1B	1.6986756	16.25236732	4.93E-14	5.73E-13
DONSON	1.3709075	17.14311226	2.73E-13	3.11E-12
TUBG1	1.0481183	19.30842173	2.86E-13	3.21E-12
POLR2G	0.902429	19.73095019	4.98E-13	5.50E-12
PSMD3	0.8884042	19.78547021	5.08E-13	5.53E-12
RFC2	1.0619402	18.79982733	5.37E-13	5.75E-12
EEF1A1	-1.030023	23.65664026	2.08E-12	2.19E-11
DHX37	0.760453	17.08651667	2.14E-12	2.22E-11
HAUS8	0.9914301	15.7658572	2.96E-12	3.03E-11
WEE1	-1.387968	17.08274774	4.90E-12	4.93E-11
ATP6V0B	0.9603342	19.9581649	7.25E-12	7.18E-11
DDX55	0.8585525	16.70882575	7.44E-12	7.27E-11
RUVBL1	1.011209	17.94444892	8.41E-12	8.10E-11
PDRG1	1.0478295	18.49086624	8.58E-12	8.14E-11
PFDN6	1.0976021	18.62236296	1.18E-11	1.11E-10
PSMB2	0.7887399	19.52673329	1.59E-11	1.47E-10
POLA2	1.0553745	16.85113392	1.91E-11	1.74E-10
RAE1	0.7571597	17.58070836	2.61E-11	2.34E-10
TOMM40	1.0175217	19.41602633	2.66E-11	2.36E-10
VPS25	0.7572079	19.22158721	3.05E-11	2.67E-10
MCM7	1.097204	19.89962288	3.41E-11	2.94E-10
PREB	0.739686	19.05909096	3.58E-11	3.05E-10
DDX56	0.7325392	19.15702777	3.76E-11	3.17E-10
ATP5MF	0.9834683	20.33849446	4.04E-11	3.37E-10
RPA3	1.0697494	17.63841849	8.79E-11	7.22E-10
MCM5	1.1830426	18.17824417	8.93E-11	7.26E-10
GPS1	0.7525967	19.07067847	1.06E-10	8.50E-10
BUB3	0.7192185	18.18344147	2.51E-10	1.99E-09
GINS4	1.6068951	14.77633106	3.45E-10	2.70E-09
POLE	0.9649307	16.38105311	3.56E-10	2.76E-09
ETF1	-0.750164	19.14161605	3.68E-10	2.82E-09
DKC1	0.844359	19.06717286	3.85E-10	2.92E-09
INCENP	1.2183125	17.2203332	4.84E-10	3.62E-09
SNRPF	1.0500622	19.05532136	6.10E-10	4.47E-09
SNRPC	0.8096768	20.60589313	6.73E-10	4.88E-09
PAFAH1B1	-0.605086	18.29841102	8.58E-10	6.15E-09
COPZ1	0.5916974	20.00489449	8.94E-10	6.35E-09
NOP2	0.8849456	18.29588722	9.19E-10	6.45E-09
GINS3	1.1572269	15.49418001	1.08E-09	7.45E-09
SPDL1	0.9707814	16.04069411	1.08E-09	7.45E-09
ILF2	0.7790538	21.18727349	1.64E-09	1.10E-08
CPSF3	0.6755723	18.46409204	1.65E-09	1.10E-08
PSMB3	0.9459693	21.61614037	1.89E-09	1.25E-08
RUVBL2	0.8029544	19.5625831	2.29E-09	1.50E-08
LSM11	-0.970621	14.96169099	3.45E-09	2.23E-08
TOPBP1	0.8788679	17.4381252	4.39E-09	2.82E-08
THOC3	0.99007	16.62123667	5.20E-09	3.30E-08
MASTL	0.9404094	17.08002511	6.19E-09	3.90E-08
CPSF4	0.7313021	17.56155294	6.59E-09	4.11E-08
PABPN1	0.7854643	19.57969163	7.06E-09	4.35E-08
ANAPC11	0.8893272	19.13527139	7.11E-09	4.35E-08
SLC39A7	0.8469647	20.76141587	7.55E-09	4.57E-08
PSMA5	0.687143	18.92197438	7.59E-09	4.57E-08
DBR1	0.6386436	16.99035299	7.79E-09	4.64E-08
TUBB	0.8694878	22.71154933	8.03E-09	4.75E-08

DDX24	-0.686423	18.34645013	8.21E-09	4.81E-08
LSM8	0.6258558	17.01141749	8.48E-09	4.93E-08
PRIM1	1.1857072	16.53545101	9.83E-09	5.66E-08
DDX49	0.73548	18.92782577	1.31E-08	7.48E-08
CENPW	1.4726612	18.83865643	1.91E-08	1.08E-07
CNOT3	0.6285266	17.98750943	2.23E-08	1.25E-07
EXOSC4	1.0306519	19.01308283	2.39E-08	1.33E-07
LSM7	0.9749655	19.67686704	2.74E-08	1.51E-07
CPSF1	0.7386648	18.92279922	2.95E-08	1.61E-07
PSMD14	0.6902806	17.7747936	3.36E-08	1.83E-07
HAUS5	0.8517093	17.10400035	3.44E-08	1.86E-07
SNRPD1	0.7831489	18.35493131	4.06E-08	2.17E-07
SEC13	0.7656536	19.22593494	4.10E-08	2.17E-07
PAM16	0.9288902	16.58792327	4.81E-08	2.53E-07
RFC3	1.1016219	17.02446824	6.01E-08	3.12E-07
ERH	0.6025336	20.72640892	6.27E-08	3.23E-07
MED8	0.6526205	18.73795764	6.33E-08	3.23E-07
NOC4L	0.7857381	18.4864836	7.70E-08	3.90E-07
ZNHIT2	0.9632562	18.04191223	8.02E-08	4.04E-07
CCT5	0.909231	20.01157305	8.48E-08	4.23E-07
MZT1	0.8082534	18.05318601	9.58E-08	4.68E-07
NFS1	0.5934298	16.99549851	9.93E-08	4.81E-07
MMS22L	0.946604	13.83735587	1.02E-07	4.93E-07
CFAP298	0.783868	17.56996161	1.10E-07	5.26E-07
RANGAP1	0.7201262	19.7395024	1.38E-07	6.49E-07
PFDN2	1.0284372	21.39695202	1.47E-07	6.86E-07
PSMA7	0.6718844	21.44924531	1.64E-07	7.61E-07
PSMB4	0.7216735	21.61651093	1.98E-07	9.15E-07
CSE1L	0.686669	19.83305957	2.33E-07	1.07E-06
MCM3	0.8485507	19.99629974	2.58E-07	1.17E-06
BANF1	0.6600934	21.09171243	2.71E-07	1.23E-06
SS18L2	0.7838921	18.64799687	2.96E-07	1.32E-06
DPAGT1	0.5962434	17.76505778	3.84E-07	1.70E-06
EIF6	0.706781	21.62775157	4.21E-07	1.85E-06
PSMA4	0.6019411	19.02698452	4.89E-07	2.14E-06
POP5	0.7166036	18.14393821	5.40E-07	2.33E-06
SF3B4	0.6013305	20.37647542	6.44E-07	2.77E-06
ATP6V1F	0.5992405	21.69312544	6.53E-07	2.78E-06
SMG5	0.5926614	19.2833139	7.52E-07	3.17E-06
CDC123	0.6002175	19.61658538	7.58E-07	3.17E-06
PSMD11	0.6824426	18.84902639	7.61E-07	3.17E-06
POLR1C	0.6686751	18.21756875	7.91E-07	3.27E-06
MCM6	0.938416	18.40912591	8.19E-07	3.35E-06
SNRPD3	0.610495	20.08179061	8.21E-07	3.35E-06
POLR3K	0.68086	17.79744582	8.49E-07	3.45E-06
SNRNP70	0.7126616	20.29215637	8.83E-07	3.56E-06
TRMT112	0.6443171	21.12610709	1.01E-06	4.04E-06
CENPN	1.0259327	16.53742614	1.06E-06	4.22E-06
COPE	0.622709	20.18031581	1.17E-06	4.62E-06
BRIX1	0.716575	17.97975647	1.21E-06	4.75E-06
LSM5	0.6178409	17.62781855	1.27E-06	4.95E-06
RPP40	0.7548489	16.82798946	1.32E-06	5.09E-06
PRPF31	0.6098685	19.39259869	1.45E-06	5.58E-06
ARMC7	0.7330439	17.54426386	1.47E-06	5.59E-06
NOP56	0.6379083	19.24182719	1.62E-06	6.13E-06
NAA10	0.7860582	17.89054142	1.73E-06	6.46E-06
WDR74	0.6427227	17.59483717	1.75E-06	6.51E-06
PSMB5	0.5924796	20.72805112	1.89E-06	6.99E-06

LSM2	0.6866078	19.97033427	1.94E-06	7.16E-06
NUTF2	0.6497128	19.66390576	1.97E-06	7.18E-06
MYC	-1.769037	19.09679459	1.97E-06	7.18E-06
HSPE1	0.6784442	20.11527752	2.67E-06	9.41E-06
LRR1	0.7721102	16.67959105	2.97E-06	1.04E-05
EEF2	-0.637794	23.40647412	3.31E-06	1.15E-05
YKT6	0.6023966	19.5679331	4.10E-06	1.40E-05
CDK7	0.6156118	17.87404382	5.48E-06	1.87E-05
TUT1	0.6074093	16.58708803	5.70E-06	1.91E-05
SF3A2	0.5953737	19.42916949	6.42E-06	2.12E-05
SMC4	1.1125097	16.94537262	8.64E-06	2.83E-05
EIF3A	-0.643592	19.42898706	9.04E-06	2.94E-05
SEC61G	0.8689203	19.78352903	9.70E-06	3.14E-05
PSMD8	0.5956198	20.33402151	1.07E-05	3.45E-05
RPP21	0.7013017	17.52887377	1.50E-05	4.69E-05
PSMG4	0.711176	15.32627716	1.75E-05	5.40E-05
TIMM10	0.633735	19.75742721	2.48E-05	7.55E-05
HYOU1	0.6795702	18.97868741	2.54E-05	7.71E-05
POLR1B	0.590407	16.60976071	3.00E-05	9.01E-05
HAUS1	0.6658171	17.77339867	3.05E-05	9.15E-05
HSPA5	0.5894163	21.9729654	3.68E-05	0.000107566
IPO7	-0.642489	18.63010563	3.94E-05	0.000113907
SNRPD2	0.597323	21.51416218	4.78E-05	0.000137066
TIGD1	0.7452222	15.5854456	7.64E-05	0.00020936
POLD2	0.6003604	19.4442588	0.000141842	0.000374909
HAUS7	0.7399553	14.38071389	0.000403567	0.000996353
KRT8	1.3972076	22.51672143	0.000501972	0.001217011
CCDC84	0.6654964	16.26259135	0.001115431	0.002506002
SMC2	0.6506946	16.70664223	0.00168662	0.003655248
POLR2F	-1.545409	8.089182436	0.005414378	0.010577654
LTO1	0.6145477	16.00051613	0.010769942	0.019887509
